# No difference in 5‐year survivorship between cemented versus cementless total knee arthroplasty in a cohort of 5266 patients using a deep‐dish mobile bearing implant

**DOI:** 10.1002/ksa.12668

**Published:** 2025-04-08

**Authors:** Ophélie Manchec, Emilie Bérard, Regis Pailhé, Sébastien Lustig, Etienne Cavaignac

**Affiliations:** ^1^ Service de Chirurgie Orthopédique et Traumatologie, hôpital Pierre‐Paul Riquet, CHU Purpan Toulouse France; ^2^ Service d'Épidémiologie Clinique et de Santé Publique, CHU de Toulouse, CERPOP, Inserm, Université de Toulouse III Paul Sabatier Toulouse France; ^3^ Service de Chirurgie Orthopédique, Clinique Aguiléra, Ramsay Santé Biarritz France; ^4^ Service de Chirurgie Orthopédique, Hopital de la Croix‐Rousse Lyon France

**Keywords:** cement, cementless, mobile bearing, prothesis design, survival rate, total knee arthroplasty

## Abstract

**Purpose:**

The best fixation method for total knee arthroplasty (TKA) remains controversial. The aim of this study is to compare the effect of cemented and cementless fixation on prosthesis survivorship. Our primary hypothesis is that there is no difference in survivorship between cemented and cementless TKA. Our secondary hypothesis is that there is no difference in aseptic revisions and functional outcomes between cemented and cementless TKA at mid‐term follow‐up.

**Methods:**

A multicentre retrospective study was done using data collected prospectively in a large cohort. The same deep‐dish mobile bearing design was used for both cemented and cementless TKA. Patients were divided into two groups according to the fixation method. The survival rate between cemented and cementless TKA was compared. Functional outcomes were collected preoperatively and at the 5‐year follow‐up.

**Results:**

Of the 5266 primary TKA included, 4549 were cementless, and 717 were cemented. At 5 years, there was no significant difference between the survivorship of the cementless (98.7% [95% confidence interval, CI: 98.2–99.1]) and cemented TKA (97.6%, [95% CI: 94.1–99.1]) (*p* = 0.468). There was no significant difference in the surgery‐free survival at 5 years between cementless (95.8% [95% CI: 94.9–96.5]) and cemented TKA (95.5% [95% CI: 92.1–97.5]) (*p* = 0.508) as well as in aseptic revision: cementless (96.9% [95% CI: 96.2–97.5]) and cemented TKA (97.5 [95% CI: 95.5–98.6]) (*p* = 0.355). There was no significant difference in the functional outcomes at 5 years.

**Conclusion:**

There was no observed difference in survivorship between cemented and cementless TKA at 5 years in this cohort of 5266 patients. Additionally, rates of reoperation and aseptic revision were similar across both fixation methods, and clinical outcomes did not differ significantly. Therefore, it may be suggested that cementless fixation is a safe option for primary TKA.

**Level of Evidence:**

Level III.

**Abbreviations**
BMI: body mass index
C: cemented
CI: confidence interval
HKA: hip–knee–ankle angle
IKS: International Knee Score
mFA: mechanical femoral axis
mTA: mechanical tibial axis
NC: cementless
TKA: total knee arthroplasty

## INTRODUCTION

Given the increasing number of total knee arthroplasty (TKA) being performed because of population growth and the broadening of indications [18, 20], enhancing the survival rate of primary TKA is a considerable challenge. The most common reason for failure is aseptic loosening, followed closely by infection [8, 17, 33]. This makes the fixation mode a primary concern: although cemented fixation is historically the gold standard [3, 12, 29], it is also a major topic of debate. Indeed, according to Fraser et al., there is a theoretical risk of aseptic loosening with cemented fixation due to lack of biological attachment [10]. Additionally, this leverages the concept of osseointegration, derived from total hip arthroplasty, aiming to achieve durable biological fixation, provided by cementless coatings. After the failure of the first generation of cementless implants [4, 28], the emergence of new implant materials such as porous metals and hydroxyapatite coatings, which have been shown to improve outcomes and implant survival [1, 5, 7, 15], has sparked renewed debate.

Despite these advances, the debate over the optimal mode of fixation in TKA persists, particularly given the difficulty of doing highly powered studies to compare cemented and cementless options, since they both have excellent survivorship [31]. Many of these studies suffer from methodological limitations such as small sample sizes, non‐comparable study groups, or inconsistent measures across different implant designs [9]. This study aims to address some of these gaps by comparing the survival rate and functional outcomes (International Knee Score [IKS], flexion) at the 5‐year follow‐up, in a large cohort of patients, using the same deep‐dish mobile bearing implant design for both cemented and cementless TKA systems. Our primary hypothesis is that there is no difference in survivorship between cemented and cementless TKA. Our secondary hypothesis is that there is no difference in aseptic revisions or functional outcomes between the two fixation methods at mid‐term follow‐up.

## MATERIALS AND METHODS

### Patients

This study is a multicentre retrospective cohort analysis, utilizing data from the Amplitude® industrial arthroplasty database, which was collected prospectively by surgeons on patients who underwent TKA with the SCORE I implant for primary osteoarthritis. The database was used under the authorization of the CNIL and registered in CliniRecord as number 1355265. Amplitude® has registered the data for the long‐term evaluation of the SCORE implant on the public platform ‘Health Data Hub’ as number F20210913151920. All data used in this study are sourced from this registry, which we managed according to CNIL standard methodology MR‐004. This study involved 15 French healthcare centres; the surgeries were performed by 16 surgeons.

**Figure 1:**
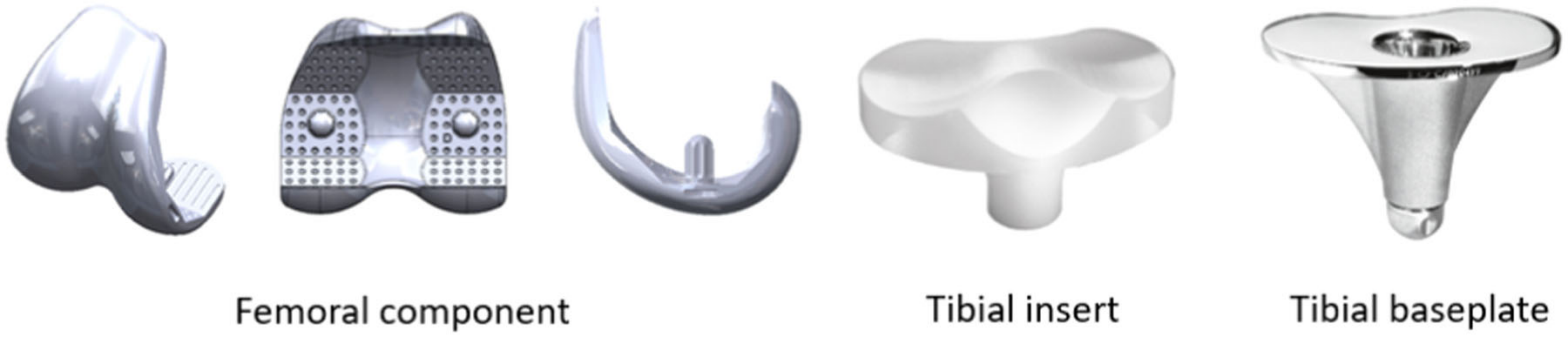
SCORE® prothesis components (Amplitude®).

The SCORE I (Amplitude®) is a congruent posterior cruciate ligament sacrificing TKA, with a rotating platform and mobile bearing (Figure 1). The design of the cemented and cementless implants is the same, with seven sizes available. The trochlear groove is angled at 6° and has a constant radius of curvature, which gives the surgeon the choice of keeping the native patella or replacing it. The tibial baseplate is stabilized by a 35 mm keel and delta wings. The implants intended for cementless fixation are coated with an 80 µm layer of plasma‐sprayed titanium and an 80 µm layer of plasma‐sprayed hydroxyapatite.

All the patients who underwent a primary TKA between March 2002 and July 2022 were included. There were no age or body mass index (BMI) restrictions. Patients could have previously undergone surgery other than UKA or TKA on the affected knee, but only primary TKAs were included. The exclusion criteria were missing data on the cementation process, TKA for inflammatory arthritis, and reconstruction prosthesis (mostly tumoral pathology). Patients who had only one of the two components cemented were also excluded from this analysis.

The cohort was separated into two groups: full cemented and full cementless. Cementation of the patellar implant was not taken into account to classify the patients into one of the two groups.

Patient characteristics are detailed in Table 1. Radiographical measurements before and after surgery are available in Table 2.

**Table 1:** Patients characteristics.

Characteristics	Cementless (*n* = 4549)	Cemented (*n* = 717)	*p* **value**	Total (*n* = 5266)
Intervention period, *n* (%)			<0.0001	
2002–2011	2113 (46.4)	468 (65.3)		2581 (49.0)
2012–022	2436 (53.6)	249 (34.7)		2685 (51.0)
Sex, *n* (%)			<0.0001	
Men	1772 (40.0)	213 (30.4)		1985 (38.7)
Women	2659 (60.0)	488 (69.6)		3147 (61.3)
Age (years)			<0.0001	
*n*/missing	4474/75	707/10		5181/85
Mean (SD)	71.0 (9.0)	74.3 (8.0)		71.4 (8.9)
Min; Max	18; 94	41; 93		18; 94
Side, *n* (%)			0.740	
Left	2184 (48.0)	349 (48.7)		2533 (48.1)
Right	2365 (52.0)	368 (51.3)		2733 (51.9)
Weight (kg)			<0.0001	
*n*/missing	4243/306	664/53		4907/359
Mean (SD)	82.0 (15.6)	78.6 (15.3)		81.5 (15.6)
Min; Max	42; 160	40; 130		40; 160
BMI (kg/m^2^)			<0.0001	
*n/*missing	4212/337	662/55		4874/392
Mean (SD)	30.3 (5.3)	29.5 (5.3)		30.2 (5.3)
Min; Max	16.0; 55.0	18.0; 53.0		16.0; 55.0
ASA, *n* (%)			<0.0001	
1	416 (10.6)	73 (10.9)		489 (10.6)
2	2396 (60.8)	473 (70.6)		2869 (62.3)
3	1105 (28.1)	121 (18.1)		1226 (26.6)
4	20 (0.5)	1 (0.1)		21 (0.5)
5	1 (0.0)	2 (0.3)		3 (0.1)
Approach, *n* (%)			<0.0001	
Antero‐medial	3843 (91.4)	637 (96.1)		4480 (92.1)
Antero‐lateral	360 (8.6)	26 (3.9)		386 (7.9)
Tourniquet, *n* (%)			0.032	
Yes	252 (7.1)	17 (4.3)		269 (6.9)
No	3274 (92.9)	380 (95.7)		3654 (93.1)
Navigation, *n* (%)			<0.0001	
Yes	2537 (55.8)	476 (66.4)		3013 (57.2)
No	2012 (44.2)	241 (33.6)		2253 (42.8)
Patellar procedure, *n* (%)			<0.0001	
Yes	1637 (36.0)	567 (79.1)		2204 (41.9)
No	2912 (64.0)	150 (20.9)		3062 (58.1)
Patellar type, *n* (%)			<0.0001	
Cementless inlay patella	476 (29.1)	31 (5.5)		507 (23.0)
Cemented inlay patella	489 (29.9)	34 (6.0)		523 (23.7)
Resurfaced cemented patella	672 (41.1)	502 (88.5)		1174 (53.3)

Abbreviations: ASA, American Society of Anaesthesiologists; BMI, body mass index; Max, maximum; Min, minimum; SD, standard deviation.

**Table 2:** Radiological measurements before and 1 year after surgery.

Radiological measurements	Cementless TKA	Cemented TKA	*p* **value**	Total
Preoperative HKA (°)			0.001	
*n*/missing	3261/1288	475/242		3736/1530
Mean (SD)	176.7 (5.2)	177.5 (5.5)		176.8 (5.3)
Post‐operative HKA (°)			0.001	
*n*/missing	1691/2858	313/404		2004/3262
Mean (SD)	179.3 (2.3)	178.9 (2.1)		179.2 (2.3)
Preoperative mFA (°)			<0.0001	
*n*/missing	2924/1625	434/283		3358/1908
Mean (SD)	91.3 (2.9)	94.6 (3.6)		91.8 (3.2)
Post‐operative mFA (°)			<0.0001	
*n*/missing	926/3623	107/610		1033/4233
Mean (SD)	90.3 (1.8)	94.3 (3.6)		90.7 (2.4)
Preoperative mTA (°)			0.151	
*n*/missing	2631/1918	284/433		2915/2351
Mean (SD)	87.2 (3.8)	87.5 (5.2)		87.2 (4.0)
Post‐operative mTA (°)			0.129	
*n*/missing	776/3773	47/670		823/4443
Mean (SD)	89.8 (1.6)	89.4 (1.7)		89.8 (1.6)

Abbreviations: HKA, hip–knee–ankle angle; mFA, mechanical femoral angle; mTA, mechanical tibial angle; SD, standard deviation; TKA, total knee arthroplasty.

### Methods

Preoperative data and post‐operative data, as well as data at the last follow‐up visit, were collected prospectively. The survival rate was defined as the time (in months) from the date of surgery until the first surgical revision of the TKA (partial or total implant change or explanation), all causes combined (implant loosening, patella, infection, fracture, stiffness, pain, implant dislocation, etc.). Patients were censored at their date of death or last follow‐up visit, if no event occurred.

Secondary outcomes included surgery‐free survival, defined as any surgery involving the same knee, regardless of whether the implants were revised or not. Another outcome was survival without aseptic revision, defined as a partial or total revision of the TKA, excluding revision due to infection.

The International Knee Society (IKS) score [25] was collected before the surgery and during the clinical reassessment until the last follow‐up. The knee's flexion range [21] was measured in degrees before, during and after the surgery. Only the results at 5 years post‐surgery were included for the statistical analysis.

Conventional knee radiographs were taken. The hip–knee–ankle angle (HKA), mechanical femoral angle (mFA) and mechanical tibial angle (mTA) were measured manually in degrees before the surgery and by the surgeon using imaging software 1 year after the surgery.

### Statistical analysis

For the primary end point, a sample size of 4549 in the cementless group and 717 in the cemented group achieves 80% power to detect equivalence with a margin of ±2%, a reference group proportion expected at 97% and an actual difference fixed to 0. The significance level is 0.05. Before the analyses, verification of missing, aberrant or inconsistent data was conducted. After corrections, the database was locked. The analysis was performed on the locked database.

The patient characteristics were summarized using the appropriate descriptive statistics according to the type of variable. Descriptive statistics included mean with SD and minimum–maximum for continuous variables and number of non‐missing observations with frequency (%) for categorical variables.

For the analysis of survival end points, Kaplan–Meier curves were generated together with 95% confidence intervals (CIs) and compared, first using the log‐rank test and then using a Cox model adjusted for age, sex, patellar procedure, BMI and period (2002–2011 vs. 2012–2022). Finally, propensity score matching (PSM) was used to adjust for potential baseline differences between cemented versus cementless TKA. A multivariate logistic regression model was generated to estimate a propensity score for each patient to receive cemented or cementless TKA. Covariates were the time period, age, sex, BMI, American Society of Anesthesiologists score, approach, tourniquet, navigation, patellar procedure and patellar type. The performance of the model was estimated with the c‐statistic (0.84 [95% CI: 0.83–0.86]). The mean propensity score was 0.399 (±0.256) in patients with cemented TKA (*N* = 717) and 0.095 (±0.130) in patients with cementless TKA (*N* = 4549). Based on the propensity score, 498 patients with cemented TKA were matched with 498 patients with cementless TKA (708 with a precision of 0.0001, 42 with a precision of 0.001, 102 with a precision of 0.01 and 144 with a precision of 0.1). The mean propensity score was the same in patients with cemented and cementless TKA (0.296 ± 0.234) in the matched sample. Survival rates before revision, surgery‐free and infection‐free survival rates were compared between cemented and cementless TKA in the subgroup of PSM subjects.

Student's *t* test was used to compare the distribution of continuous secondary end points (or Mann–Whitney's test when the normality or homoscedasticity assumptions were rejected). The change in IKS and flexion at 5 years was also compared between groups after adjusting for preoperative value, age, sex, patellar procedure, BMI and period using a linear regression model.

All reported *p* values were two‐tailed, and the significance threshold was <0.05.

Statistical analyses were performed using STATA software 18.0 (STATA Corp.).

## RESULTS

**Figure 2:**
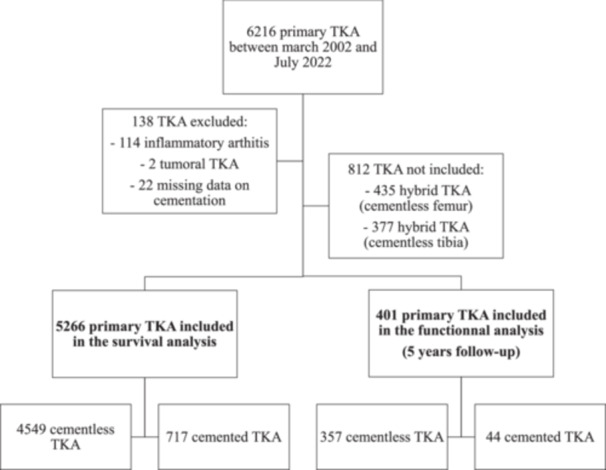
Flowchart of the inclusion of the patients. TKA, total knee arthroplasty.

The survival analysis comprised 5266 TKA, 4549 (86.4%) in the cementless group and 717 (13.6%) in the cemented group. A total of 1398 (26.5%) patients achieved a 5‐year follow‐up. For the functional outcomes, 401 patients who attained the 5‐year follow‐up were included in the secondary analysis. Details of the inclusion criteria are provided in the flowchart (Figure 2).

### Primary end point—Survival rate

**Figure 3:**
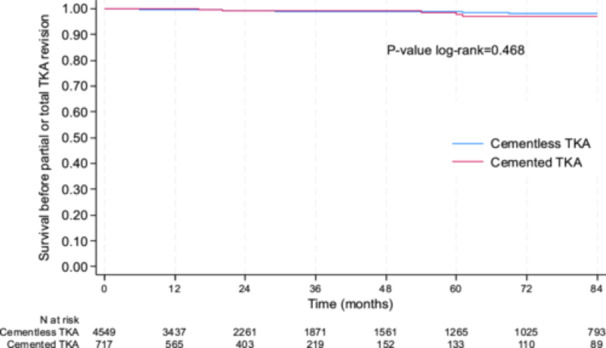
Revision‐free survival rate between cemented and cementless TKA. TKA, total knee arthroplasty.

Overall, the cumulative survival rate between cementless and cemented TKA was not statistically different (Figure 3). At 5 years, the survival rate before revision was 98.7% (95% CI: 98.2–99.1) in the cementless group and 97.6% (95% CI: 94.1–99.1) in the cemented group (*p* = 0.468). Results were the same when adjusted for age, sex, BMI, patellar procedure and period (2002–2011 vs. 2012–2022) (*p* = 0.453) and in the sensitivity analysis conducted in the subgroup of PSM subjects. Indeed, at 5 years, the survival rate before revision was 99.3% (95% CI: 97.9–99.8) in the cementless group and 98.2% [95% CI: 94.7–99.4] in the cemented group (*p* = 0.448). The ±2% equivalence margin was not reached.

**Figure 4:**
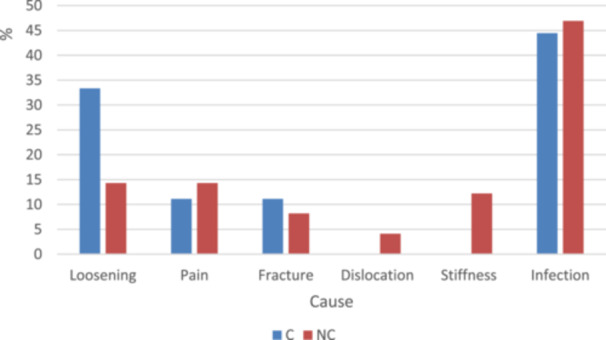
Distribution of causes of revision by fixation type. C, cemented; NC, cementless.

The various reasons for revision are listed in Table 3, and the distribution by type of fixation is in Figure 4. The primary reason for the revision was an infection in both groups (44.4% in the cemented group and 46.9% in the cementless group), followed by loosening (33.3% in the cemented group and 14.3% in the cementless group) and pain in the cementless group (14.3%) (*p* = 0.763).

**Table 3:** Reason for revision by type of cementation.

	Cemented TKA	Cementless TKA		Total
	*n* = 9 (15.5)	*n* = 49 (84.5)	*p* **value**	*n* = 58 (100.0)
Cause, *n* (%)			0.763	
Loosening	3 (33.3)	7 (14.3)		10 (17.2)
Pain	1 (11.1)	7 (14.3)		8 (13.8)
Fracture	1 (11.1)	4 (8,2)		5 (8.6)
Dislocation		2 (4.1)		2 (3.4)
Stiffness		6 (12.2)		6 (10.3)
Infection	4 (44.4)	23 (46.9)		27 (46.6)

Abbreviation: TKA, total knee arthroplasty.

### Secondary end points—Surgery‐free survival rate, survival rate without infection and functional outcomes

**Figure 5:**
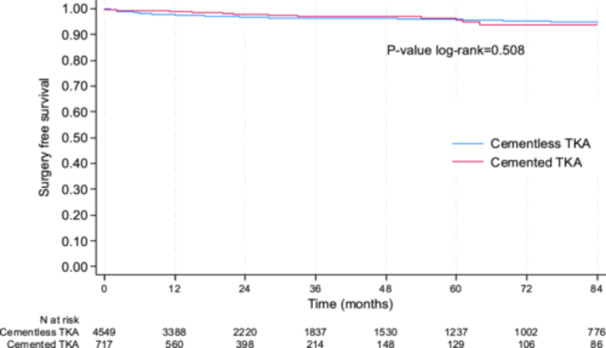
Surgery‐free survival rate between cementless and cemented total knee arthroplasty (TKA).

The cumulative surgery‐free survival rate was not statistically different between cementless and cemented TKA (Figure 5), with a survival rate at 5 years of 95.8% (95% CI: 94.9–96.5) and 95.5% (95% CI: 92.1–97.5) (*p* = 0.508), respectively. Results were the same when adjusted for age, sex, patellar procedure, BMI and period (2002–2011 vs. 2012–2022) (*p* = 0.899) and in the sensitivity analysis conducted in the subgroup of PSM subjects. Indeed, at 5 years, the surgery‐free survival rate was 97.4% (95% CI: 95.1–98.6) in the cementless group and 95.1% (95% CI: 91.5–97.2) in the cemented group (*p* = 0.285).

**Figure 6:**
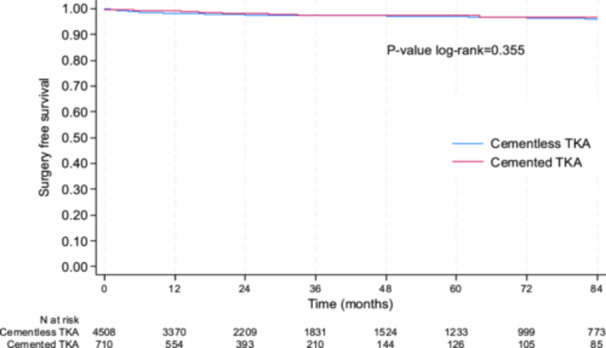
Surgery‐free survival rate between cemented and cementless total knee arthroplasty (TKA), infection excluded (*N* = 54).

All surgery for infections excluded (*N* = 54), the cumulative surgery‐free survival rate was not statistically different between cementless and cemented TKA (Figure 6), with a survival rate at 5 years of 96.9% (95% CI: 96.2–97.5) and 97.5% (95% CI: 95.5–98.6) (*p* = 0.355), respectively. Results were the same when adjusted for age, sex, patellar procedure, BMI and period (2002–2011 vs. 2012–2022) (*p* = 0.653) and in the sensitivity analysis conducted in the subgroup of PSM subjects. Indeed, at 5 years, the surgery‐free survival rate without infection is 98.8% (95% CI: 96.7–99.6) in the cementless group and 96.8% (95% CI: 94.1–98.3) in the cemented group (*p* = 0.149).

There was no significant difference in the improvement in functional outcomes at 5 years (Table 4). The IKS was 182 (±19) in the cementless group, reflecting an improvement of 84 points (±24) from the preoperative score, and 175 (±29) in the cemented group (*p* = 0.322), an improvement of 75 points (±30) from the preoperative score (*p* = 0.060). Results were the same when adjusted for preoperative IKS, age, sex, patellar procedure, BMI and period (2002–2011 vs. 2012–2022) (*p* = 0.099).

**Table 4:** Functional outcomes before surgery and at 5 years [34].

Measure	Cementless TKA	Cemented TKA	*p* **value**	Total
Preoperative flexion (°)			<0.0001	
*n*/missing	4316/233	685/32		5001/265
Mean (SD)	113.0 (13.8)	108.0 (13.2)		112.3 (13.8)
Flexion at 5 years (°)			0.002	
*n*/missing	357/4192	44/673		401/4865
Mean (SD)	118.5 (11.4)	112.7 (13.3)		117.8 (11.8)
Change in flexion at 5 years (post‐ to preoperative)			0.315	
*n*/missing	337/4212	39/678		376/4890
Mean (SD)	5.0 (13.2)	7.3 (16.7)		5.2 (13.6)
Preoperative IKS			<0.0001	
*n*/missing	3640/909	571/146		4211/1055
Mean (SD)	97.4 (21.1)	90.0 (21.4)		96.4 (21.3)
IKS at 5 years			0.322	
*n*/missing	279/4270	34/683		313/4953
Mean (SD)	182.3 (19.0)	174.6 (28.9)		181.5 (20.4)
Change in IKS at 5 years (post‐ to preoperative)			0.060	
*n*/missing	243/4306	26/691		269/4997
Mean (SD)	84.5 (24.3)	74.8 (29.9)		83.6 (25.0)

Abbreviations: IKS, International Knee Score; SD, standard deviation.

Knee flexion was measured at 118 (±11) in the cementless group, an improvement of 5° (±13) and 113 (±13) in the cemented group (*p* = 0.002), an improvement of 7° (±17) from the preoperative flexion (*p* = 0.315). Results were the same when adjusted for preoperative flexion, age, sex, patellar procedure, BMI and period (2002–2011 vs. 2012–2022) (*p* = 0.328).

## DISCUSSION

The most important finding of this study is that there were no differences in the survivorship between cemented and cementless TKA in a cohort of more than 5000 patients using the same TKA design, which is consistent with previous publications featuring smaller cohorts [22, 36]. Both fixation methods produced similar results in terms of reoperation rates, infection‐free survival and functional outcomes.

This study contributes significantly to the existing literature with its substantial patient cohort, providing robust support for the findings of previous studies, even though the significance threshold was not reached due to the excellent survival rates of TKA implants. By comparing two fixation types using the same TKA design, our research addresses biases related to implant design and mechanical properties. Specifically, this research offers a detailed comparison between cemented TKA and cementless TKA coated with plasma‐sprayed titanium and hydroxyapatite. Specifying the cementless coating type in our study enhances its comparability with other research using the same coating, thereby adding significant value to our analysis of this type of cementless TKA [31].

The primary end point of cumulative survival rate showed similar clinical results with no statistical difference between the two groups over 5 years. We chose 5‐year survivorship as the end point to maximize the statistical power of our results. However, the survival curves demonstrate similar long‐term survival between the two groups, with a low revision rate. This finding is significant as it indicates that both cemented and cementless TKA can deliver durable outcomes over a 5‐year timeframe. This is consistent with previous publications. Based on the Dutch Arthroplasty Register with 201,211 cases, Quispel et al. also showed that cemented and cementless TKAs had comparable short‐ and mid‐term revision rates [32]. In their randomized controlled trial, Hannon et al. had a 100% survival rate with no significant differences between cemented and cementless fixation in 127 patients [14]. Fricka et al. found a survival rate before the revision of 95.9% and 95.3% for cemented and cementless TKA, respectively, in 85 patients at 5 years [11], with a TKA using a press‐fit design, like in our study. Those results are well documented in the literature, with robust meta‐analysis studies showing similar survival rates before revision [24, 31, 35, 36].

The analysis of revision causes between cemented and cementless TKA groups revealed a broadly comparable distribution, with no statistically significant differences (*p* = 0.763), reinforcing the notion that both fixation methods are viable options with comparable failure profiles. While there are small variations in the specific reasons for revision, the lack of significant differences supports the flexibility of choosing either approach depending on clinical context and surgeon preference. The leading causes of revision in this study, specifically infection and aseptic loosening, align with those commonly reported in previous publications [8, 30]. This supports the notion of similar revision profiles for both fixation methods, although the low number of revisions limits the statistical power to detect significant differences. Continued monitoring and refinement of techniques are essential to address the leading causes of revision, particularly infection and aseptic loosening, to further improve patient outcomes.

Similarly, the secondary end point of surgery‐free survival rate showed no significant differences between fixation methods, consistent with the primary end point. This result underscores the reliability of both fixation methods in terms of maintaining implant integrity and function. The exclusion of surgeries for infection and the subsequent analysis also did not show any significant differences, indicating that the mode of fixation seems not to markedly affect the risk of infection‐driven revisions. In their meta‐analysis, Onggo et al. found no significant differences in the rate of periprosthetic joint infection requiring revision surgery, even if the rate of revision for infection tended to be lower in cementless TKA than in cemented TKA (odds ratio: 0.89; 95% CI: [0.30–2.41]) [27]. These results suggest that the choice between cemented and cementless fixation does not significantly impact the risk of infection.

In terms of functional outcomes, our study revealed no significant differences in overall knee function as measured by the IKS and knee flexion at the 5‐year mark. However, the cementless group had slight but statistically better knee flexion, which suggests that cementless TKA contributes to a better range of motion post‐operatively. These findings are similar to those of Miller et al. in their matched case‐control study, with an improvement in the KSS and range of motion in the cementless group [23]. In their systematic review, Arnold et al. identified no difference in clinical outcomes between cemented and cementless TKA, but insisted on the many methodological biases such study could involve [2]. However, the threshold for significance varies across studies from 3.8° to 8.8° [6, 21, 34]; thus, the clinical impact of a 5.5° difference in flexion should be viewed with caution.

Cost studies seem to imply that cementless TKA is more expensive than cemented ones. In the United States, Gwam et al. found higher inpatient charges and costs but shorter mean length of stay, and higher odds of being discharged to home with cementless fixation [13]. Lawrie et al. estimated a general increased charge in the surgical procedure of $366 for cementless TKA implants over cemented TKA implants [19]. The literature indicates a significant cost associated with TKA revisions, which include extended hospital stays, especially in cases of revision for infection [16, 26]. The lack of significant differences between cemented and cementless TKAs in this study does not allow us to offset the current higher cost of cementless TKAs.

While this study provides valuable insights, it is not without limitations. The absence of significant differences in many outcomes might be influenced by the inherent study design. The fixation mode choice was based, in part, on surgeon preference and intraoperative assessment of bone quality, leading to potential selection bias and cohort heterogeneity. To minimize the impact of this bias and account for these heterogeneities, our analyses were adjusted for the main confounding factors and a propensity score was used, with no change to the results. Radiographic results are difficult to interpret and require cautious analysis due to variability in measurement methods, which may not be consistently reproducible across surgeons. Although some significant differences were found, they also should be interpreted with caution, as they have limited clinical relevance (e.g., a 3° difference in non‐standardized measurements). We can also note that the study design has led to a high number of lost follow‐up cases. Although this is offset by the final sample size, it may still introduce a bias that affects the results, particularly in the clinical outcomes analysis, where it has led to fewer exploitable data points. Conversely, this study embodies the largest comparative analysis of cementless and cemented TKA implants of the same design. As such, it allows for a more direct comparison of fixation methods, without potentially confounding differences such as component design seen in prior studies.

## CONCLUSION

There was no observed difference in 5‐year survivorship between cemented and cementless TKA in this cohort of 5266 patients. Additionally, rates of reoperation and aseptic revision were similar across both fixation methods. Clinical outcomes appeared comparable between the two fixation methods, with no significant differences. Therefore, it may be suggested that cementless fixation is a safe option for primary TKA.

## AUTHOR CONTRIBUTIONS

Data collection and article writing: Ophélie Manchec. Statistical analysis and manuscript review: Emilie Bérard. Manuscript review: Regis Pailhé. Manuscript review and final approval of the manuscript: Sébastien Lustig. Study design, manuscript review and final approval of the manuscript: Etienne Cavaignac.

## CONFLICT OF INTEREST STATEMENT

Etienne Cavaignac: Consultant for Arthrex, Amplitude and Biobank. o Sébastien Lustig: Royalties from Stryker and Smith & Nephew and institutional support from Amplitude. The remaining authors declare no conflicts of interest.

## ETHICS STATEMENT

The use of the database was conducted under the authorization of the CNIL, registered in CliniRecord under number 1355265. Amplitude® has registered the data for the long‐term evaluation of the SCORE prosthesis on the public platform ‘Health Data Hub’ under number F20210913151920. All data used in this study are sourced from this registry, which we managed according to the CNIL standard methodology MR‐004.

## Data Availability

The data sets generated and analyzed during the current study are not publicly available due to confidentiality agreements but are available from the corresponding author on reasonable request.
